# Digisonic SP^®^ Binaural cochlear implant: the coronal tunneled approach

**DOI:** 10.5935/1808-8694.20130054

**Published:** 2015-10-04

**Authors:** Guilherme Machado de Carvalho, Alexandre Caixeta Guimarães, Ivan Senis Cardoso Macedo, Lúcia Cristina Beltrame Onuki, Fabiana Danieli, Henrique Furlan Pauna, Fernando Laffitte Fernandes, Jorge Rizzato Paschoal, Walter Adriano Bianchini, Arthur Menino Castilho

**Affiliations:** a MSc in Medicine (Nova University in Lisbon) (ENT, Otologist, Course of Otorhinolaryngology, Head, and Neck, UNICAMP).; b MD (ENT Resident, Course of Otorhinolaryngology, Head, and Neck, UNICAMP).; c MD (ENT, Otologist, Course of Otorhinolaryngology, Head, and Neck, UNICAMP).; d Speech and Hearing Therapist, Course of Otorhinolaryngology, Head, and Neck, UNICAMP. (Speech and Hearing Therapist, Specialist in Cochlear Implants).; e MSc in Bioengineering, Speech and Hearing Therapist (Speech and Hearing Therapist, Specialist in Cochlear Implants, MSc in Bioengineering, EESC/USP).; f MD (ENT Resident, Course of Otorhinolaryngology, Head, and Neck, UNICAMP).; g Professor (MD, ENT, Course of Otorhinolaryngology, Head, and Neck, UNICAMP).; h MSc (ENT, Otologist, Course of Otorhinolaryngology, Head, and Neck, UNICAMP).; i PhD (ENT, Otologist, Course of Otorhinolaryngology, Head, and Neck, UNICAMP). Otology, Audiology and Implantable Ear Prostheses - Ear, Nose, Throat and Head & Neck Surgery Department. UNICAMP.

**Keywords:** cochlear implantation, deafness, hearing loss, bilateral, hearing loss, sensorineural, rehabilitation of hearing impaired

## Abstract

Cochlear implants represent a significant breakthrough in the treatment of hearing loss. Evidence indicates bilateral hearing brings significant benefits to patients, particularly when binaural hearing is offered.

**Objective:**

To describe the first case of implantation of a Digisonic SP^®^ Binaural Neurelec device in Brazil (the third implant placed in the Americas, after Mexico and Colombia) and the chosen surgical approach.

**Method:**

Description of a surgical approach.

**Results:**

The procedure was successfully completed.

**Discussion:**

The squelch effect, binaural summation, location of the sound source, and the shadow effect of the head are listed among the reasons to explain the superiority of binaural rehabilitation. Cost of treatment must be considered in the development of public health policies.

**Conclusion:**

The cost of cochlear implants has been one of the main impediments to bilateral rehabilitation. The Digisonic SP^®^ Binaural Neurelec device addresses this issue and exposes patients to less risk through a minimally invasive implantation procedure.

## INTRODUCTION

Recent studies have considered cochlear implants (CI) for the treatment of profound deafness in adults and children. The early restoration of auditory input allowed by the placement of cochlear implants has allowed patients to improve their communication skills in varying degrees^1^.

According to the World Health Organization (WHO), in 2025 there will be approximately 1.2 billion people in the world aged 60 and above^2^. The Royal National Institute for Deaf People (RNID) estimates that over 300 million people experience hearing loss today, and that this number will grow to 900 million by 2050^2^, thus significantly increasing the need for rehabilitation of subjects with hearing loss.

Cochlear implants represent a significant innovation in the fields of surgery^3^ and technology. Numerous studies have stated that the benefits of implanting cochlear devices outweigh the risks, in addition to improving the quality-of-life of implanted patients^3^.

Unilateral cochlear implants allow patients to recognize speech in silent conditions, but CI users frequently report difficulty recognizing speech in the presence of background noise and locating sound sources. Patients are increasingly interested in having binaural cochlear implants^4^, as they would be able to overcome the obstacles mentioned above.

The goal of this paper was to describe the surgical technique and the first case of a bilateral Digisonic SP^®^ Neurelec cochlear implant case done in Brazil through a subcutaneous tunnel in a coronal access, with the help of an orotracheal tube and showing the details involved in this procedure. Today, UNICAMP already has a number of cases operated, all without any complications, and we also state that some Brazilian institutions have already performed this procedure.

Today, (2013), according to Neurelec itself, such procedure had been carried out only in some countries like France, the United Kingdom, Germany, Spain, Italy, Romania, Middle East, India, Mexico, Colombia, and now, Brazil.

## METHOD

Patients had to meet the following criteria to be included in the study:

• Age above 16 years (growth of the skullcap)
• Post-lingual patients with developed speech
• Good lip reading skills
• At least two years of experience with hearing aids
• Series of historical pure-tone audiometry tests (severe/profound sensorineural hearing loss -evidence of stable audiological indicators for at least two years)
• Free-field audiometry with and without hearing aids (with little gain)
• Speech recognition test (performance under 60% with 65 dB on free field)
• Absent brainstem auditory evoked potential (BAEP) and otoacoustic emissions (OAE)
• CT scans of the ears, mastoid, and skull
• MRI scans of the ears, mastoid, and skull with assessment and reconstruction of the inner ear canal
• Psychological evaluation.

Selected patients were informed of the tests, surgery, cochlear devices, postoperative expectations, expected complications and signed an informed consent term.

### Ethical aspects

The study was approved by the institution's Ethics Committee (004/2013).

## RESULTS

This section includes a description of the patient and implantation approach.

### Surgical approach

The patient was positioned in dorsal decubitus, administered general anesthesia, and intubated. Preparation was performed as follows:

1. Antisepsis of the face, retroauricular area, hair and scalp with 2% chlorhexidine; fixation of facial nerve electrodes, and administration of one gram of intravenous cefazolin
2. Bilateral retroauricular hair removal, isolation and draping of the retroauricular area and face with Micropore™
3. Preparation and containment of the patient's hair with elastic bands; marking of the coronal area
4. Application of topical anesthesia (2% xylocaine with norepinephrine; 4 mg of lidocaine/ kg) in the retroauricular region and on the coronal suture
5. Additional antisepsis with 0.5% chlorhexidine of the retroauricular area and scalp
6. Placement of sterile draping exposing the coronal suture, retroauricular areas, and part of the face
7. Straight retroauricular incision of approximately four centimeters; dissection in planes and production of a cross-shaped flap of periosteal muscle
8. Removal of small fragments of fascia and temporal muscle for later obliteration of the cochleostomy
9. Mastoidectomy, exposing the lateral semicircular canal, the short branch of the incus, the posterior wall of the outer ear canal, the tegmen tympani, and the lateral sinus; a small amount of bone powder was collected
10. Thinning of the posterior wall of the outer ear canal; posterior tympanotomy sparing the incus buttress
11. One-millimeter cochleostomy in the anterosuperior region in relation to the round window after the identification of the round window niche
12. Steps 7 to 11 were repeated on the contralateral side
13. Coronal incision of three centimeters (on the head vertex) and dissection in planes
14. Construction of a subperiosteal tunnel to connect the coronal and retroauricular incisions with the aid of lifters, retractors and conventional forceps (bilaterally)
15. Placement of a 5 mm orotracheal tube through the tunnel described above (bilaterally) (Figure 1)

**Figure 1:**
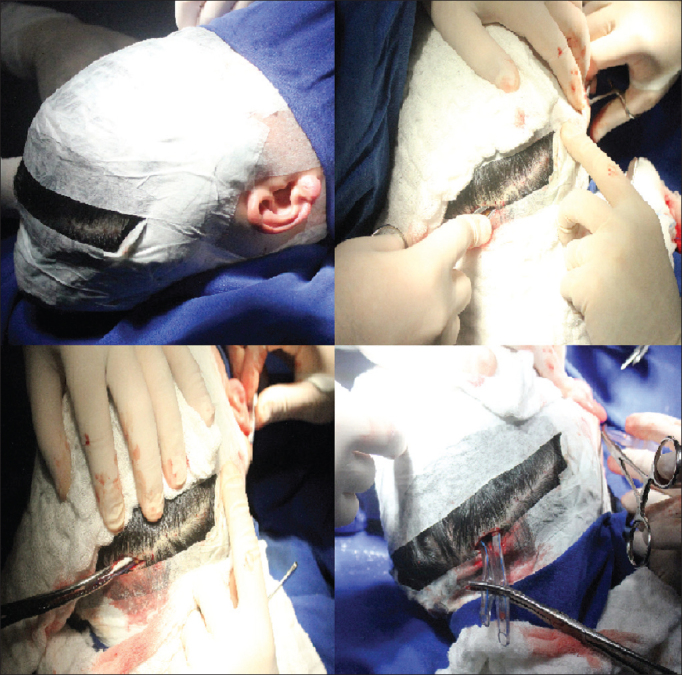
Sequence of pictures showing patient preparation and placement of 5 mm orotracheal tubes to aid electrode insertion.
16. Careful examination of hemostasis status
17. Fixation of the internal component of the binaural Neurelec Digisonic SP^®^ implant with two titanium screws on the squamous portion of the temporal bone (Figure 2)

**Figure 2:**
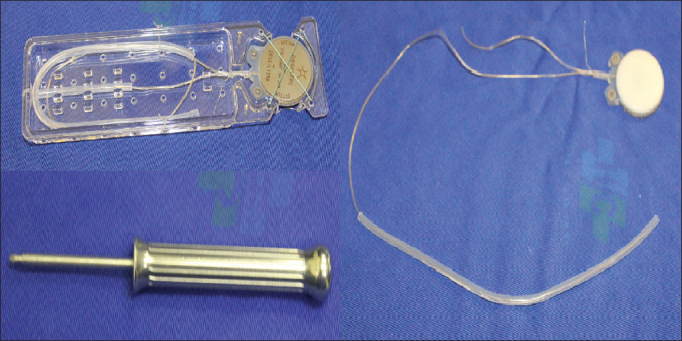
Fixation system of the internal component with titanium screws and the Neurelec Digisonic^®^ Binaural device. Note the size of the contralateral electrode.
18. Insertion of a lead through the cochleostomy with the aid of a microscope, placement of the ground electrode in the region of the zygomatic arch and positioning of the contralateral lead from within the ipsilateral orotracheal tube
19. Orotracheal tube was pulled ipsilaterally to the implant internal component through the coronal incision along with the previously positioned contralateral lead (Figure 3)

**Figure 3:**
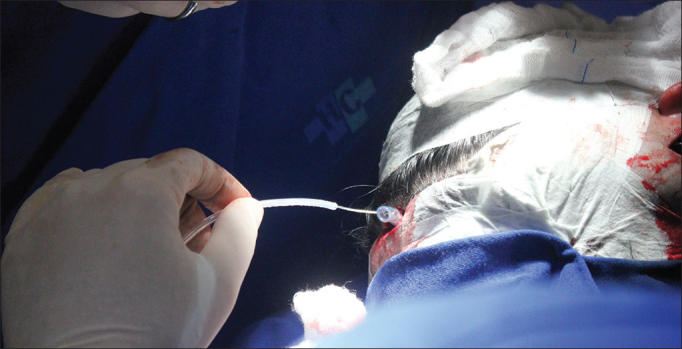
Example of how the contralateral electrode is transposed via the orotracheal tube.
20. Positioning of the contralateral lead now in the contralateral orotracheal tube
21. Orotracheal tube was pulled contralaterally to the implant through the contralateral retroauricular incision
22. Insertion of the lead through the cochleostomy with the aid of a microscope; placement of a ground electrode in the region of the zygomatic arch (contralateral side)
23. Positioning of a muscle graft around the electrode in a way to seal the cochleostomy; placement of bone powder to obliterate the posterior tympanotomy (bilaterally)
24. Sutures applied with Vicryl™ 3.0 to close the planes of the muscle periosteum flaps; closure of the coronal incision and subcutaneous stitches with nylon 4.0 on the plane of the skin (bilaterally)
25. Patient was cleaned and external compressive bandages were placed
26. Electrode impedances and brainstem auditory evoked potentials (BAEPs) were measured
27. Transorbital view x-ray images were taken to confirm the position of the intracochlear lead (Figure 4).

**Figure 4:**
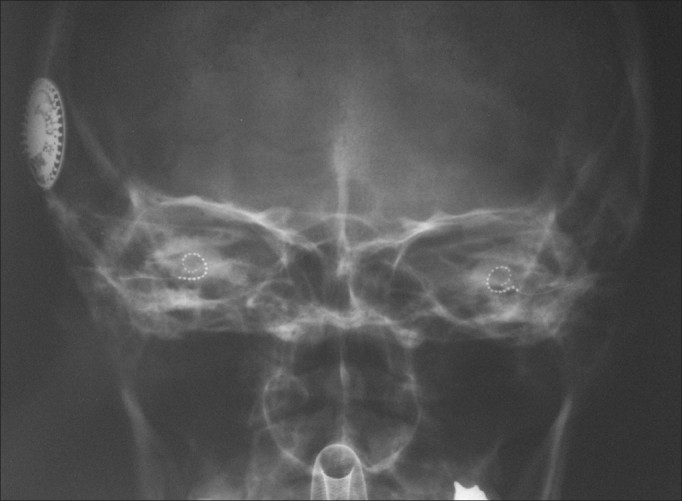
Transorbital x-ray image of the patient at the end of the procedure showing both electrodes inserted in the cochleas.

Note: The patient's head must be moved with utter care. This surgical approach was not developed by the authors of this paper; it is an adaptation from previously described procedures^5^, ^6^.

**Figure 5:**
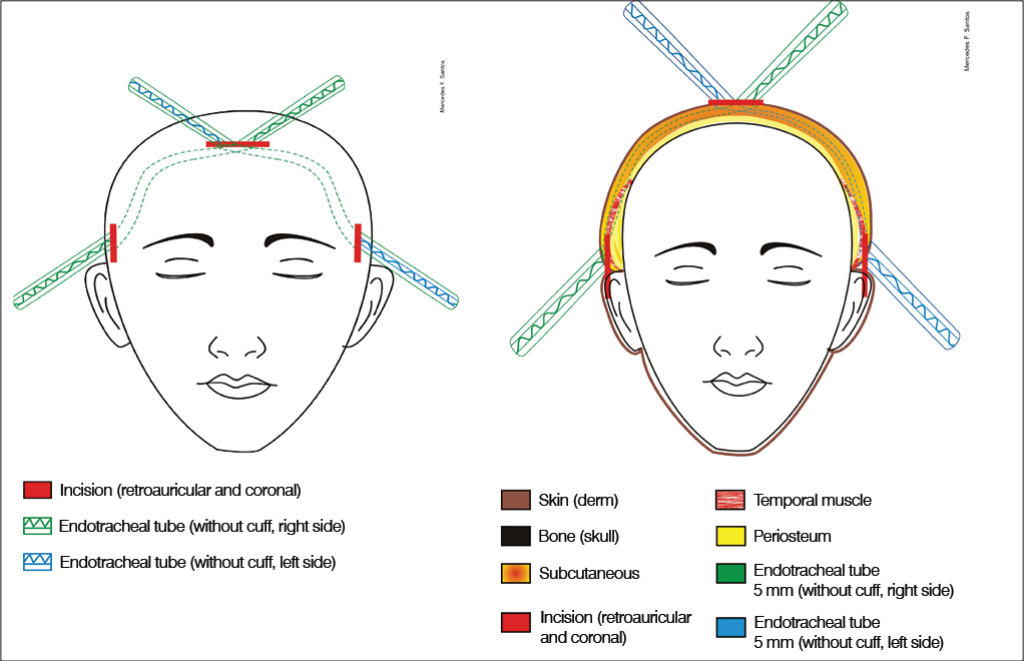
Schematic representation showing the planes on which the orotracheal tubes are placed. See legend for the involved anatomic structures.

**Figure 6:**
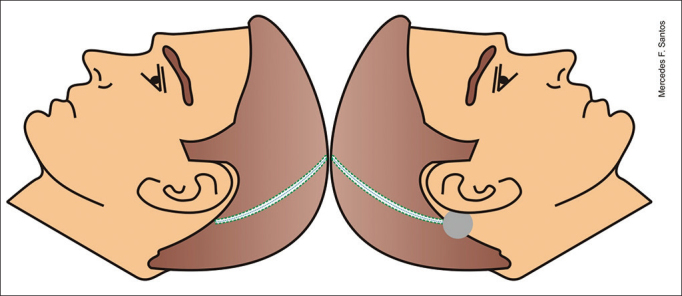
Schematic representation of the site through which the electrode is placed.

**Figure 7:**
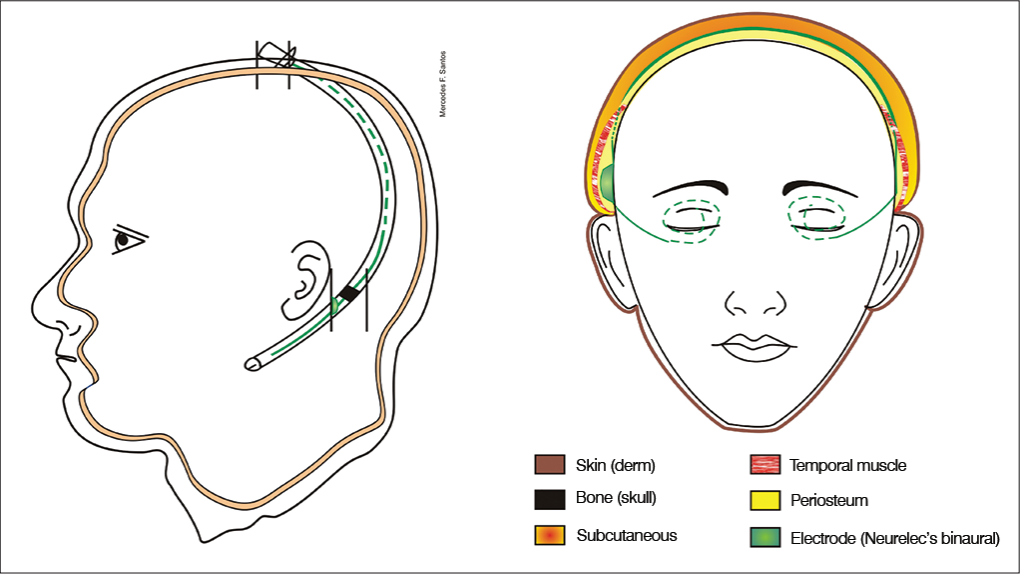
Schematic representation showing the left view of the internal component in place. The electrode is placed in the subperiosteal area in the temporal region and subcutaneously the rest of the way.

Surgery is depicted in Figure 5, Figure 6, Figure 7.

#### Impedance and BAEP measurement

**Figure 8:**
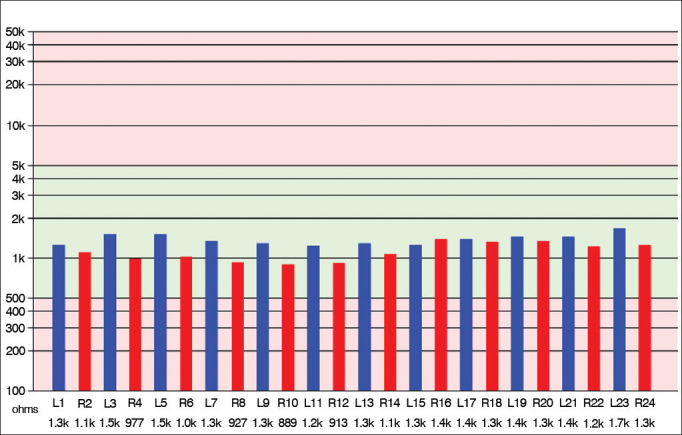
Impedance measurements of the electrodes inserted bilaterally. The bars represent the impedance values recorded for each electrode in the ipsilateral and contralateral beams depicted in blue and red. Impedance levels were within normal range*, and were under 2,000 Ohms for all tested electrodes.

**Figure 9:**
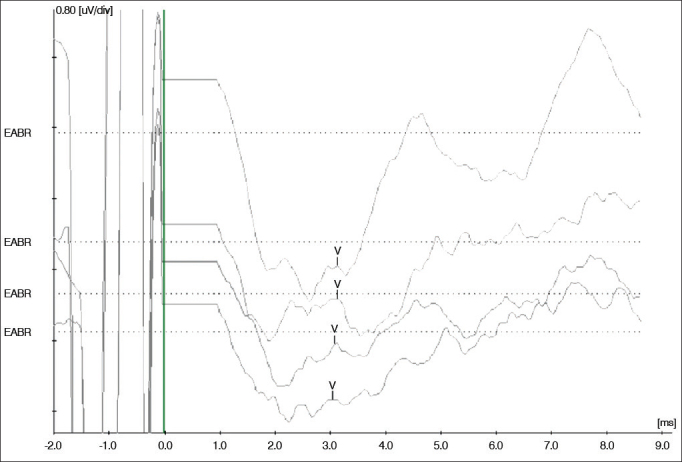
Brainstem auditory evoked potentials measured during surgery for apical and medial electrodes inserted bilaterally. From top to bottom, the measurements correspond to electrodes R24, L23, R12, and L13 respectively. Wave V was observed in all electrodes, showing the implanted device functioned properly to provide effective neural fber stimulation. Measurements made with Interface Navigator Pro and Software AEP version 7.0.0, Biologic.

Impedances and BAEPs were measured for the electrodes inserted in both cochleas through a bidirectional telemetry system. Diagnostic interface Digistim SP and software Digistim for Windows SP^®^, version 1.9.15 were used with such purpose. Measurements were carried out during and after surgery with the patient still under general anesthesia in order to check the status of the receiver-stimulator and the electrode beams, device overall function, and effectiveness of the stimuli delivered upon the peripheral auditory neural fibers in both sides (Figures 8 and 9).

#### Facial Nerve Monitoring

The facial nerve was monitored bilaterally throughout the entire procedure through two electrodes placed one on each side of the face on the frontal and zygomatic areas, in addition to ground leads (placed on the chest) and STIM + (positive pole on the sternoclavicular area). A NIM- Pulse™ (Nerve Integrity Monitor, Meditronic Xomed™) monitor was used.

#### Microscope

A CARL ZEISS GMGH S88 Microscope™ was used. The microscope was equipped with a camera and a digital video recording system to capture images of the implantation procedures.

### Device

Cochlear implant Neurelec Digisonic SP^®^ Binaural, developed by French company Neurelec S.A. in 2006 was used in this study.

**Figure 10:**
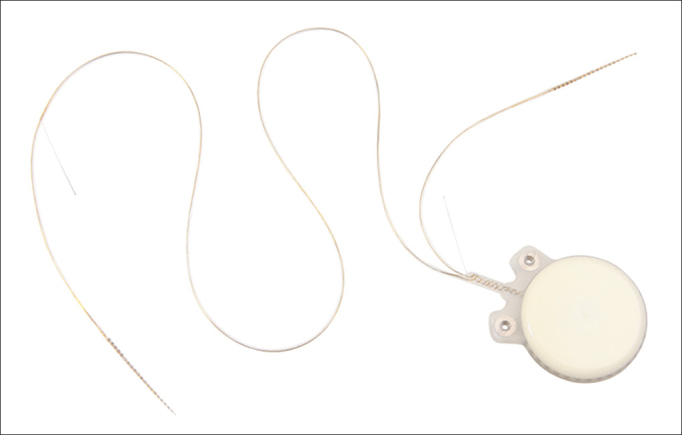
Digisonic SP^®^ Binaural cochlear implant. Receiver-stimulator and ipsilateral and contralateral electrode beams.

**Figure 11:**
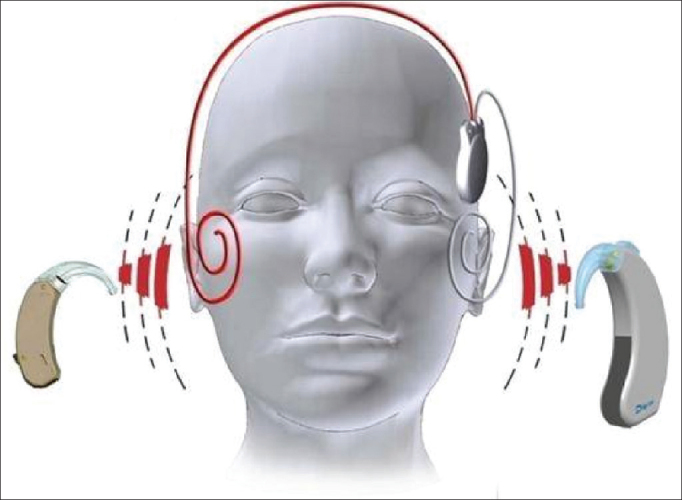
Schematic representation showing sounds acquired by the microphone of a conventional speech processor and by the contralateral microphone connected to it. Both are processed by the speech processor and sent to one single implanted receiver-stimulator, responsible for transmitting the information simultaneously to the ipsilateral and contralateral electrode beams, so they synchronously stimulate the neural fbers of both cochleas.

The device is made up of one receiver-stimulator connected to two electrode beams designed to stimulate the remaining neural fibers of both cochleas in a simultaneous, synchronous fashion (Figure 10). The receiver-stimulator is physically similar to the conventional monaural Digisonic SP^®^ implant, and allows for quick, minimally invasive implantation. Each beam has 12 leads connected to a ground electrode, adding up to 24 active stimulation channels and speeds of up to 24,000 pulses per second. A contralateral microphone is connected to a Digi^®^ SP or Saphyr^®^ SP conventional speech processor, to separately analyze the input signals from each ear and send them synchronously to the leads positioned in each cochlea, thus producing binaural hearing (Figure 11). A Widex CROS (Widex Corp, Denmark) microphone was used.

### Case

The patient was a 35-year-old woman with history of progressive hearing loss; she had been affected by severe sensorineural hearing loss for ten years. The patient had been using hearing aids in both ears for 16 years, recently with little effect.

Due to the progressive nature of her condition, she developed oral communication and acquired good lip reading skills. The patient had a family history of deafness (father, paternal uncles and aunts, siblings, grandparents).

The patient was included in the Cochlear Implantation Program of the Otology and Implantable Devices Group of the University Hospital in January of 2012.

Clinical examination, lab workup, and imaging (CT and MRI) tests failed to reveal the etiology of her condition, as all test were normal.

Her audiological assessment is described below (Tables 1 and 2).

**Table 1:** Preoperative pure-tone audiometry.

Frequency (Hz)	250	500	1000	2000	3000	4000	6000	8000
Right ear (dB)	70	85	105	115	115	120	Abs.	Abs.
Left ear (dB)	55	75	105	115	110	120	120	Abs.

Abs.: Absent.

**Table 2:** Preoperative free-field audiometry (with hearing aids).

Frequency (Hz)	250	500	1000	2000	3000	4000	6000	8000
Right ear (dB)	65	65	75	85	Abs.	Abs.	Abs.	Abs.
Left ear (dB)	75	80	70	90	Abs.	Abs.	Abs.	Abs.
AASlemAO	60	70	70	85	Abs.	Abs.	Abs.	Abs.

Abs.: Absent.

Brainstem auditory evoked potentials, transient and product distortion otoacoustic emissions were absent for both ears.

Her scores in the Ling Six-Sound test were 0% for sound perception (each ear separately and both ears together); and 0% for name and sentence recognition (each ear separately and both ears together).

## DISCUSSION

Still today, various public health care centers recommend the implantation of unilateral - instead of bilateral - cochlear devices to patients with severe to profound sensorineural hearing loss. The reasons are many, and include cost, sparing one ear for future technologies, the additional risk of a second procedure, and the lack of evidences documenting the benefits of bilateral cochlear implants^7^.

However, bilateral cochlear implants have been considered as significantly better than unilateral cochlear devices at improving speech recognition in noise^8^.

Studies also indicate that bilateral implants (two implants, one on each ear) introduce stereophonic hearing, which results in better speech recognition in noise and silence, in addition to improving sound localization^9^.

Improved sound localization in bilateral cochlear device users relies on the shadow effect of the head, on the squelch effect, and on binaural summation^10^.

The shadow effect of the head stems from the obstacle the head offers to the arrival of sound to the stimulated ear and the improvement on the signal to noise ratio. Binaural summation is the outcome of central auditory processing and represents the ability the central auditory nervous system to integrate and use the information coming from both ears. The squelch effect represents the ability of the auditory system to utilize the information sent by both ears when speech and noise are separated spatially^4^.

Although the literature on psychoacoustics has for long discussed the benefits of binaural hearing, only recently have studies shown improved speech intelligibility in bilateral implant users when compared to patients implanted with unilateral devices.

In ideal conditions, the benefits of bilateral implants may be far greater than reported. For example, the benefit is considerably greater in speech intelligibility in noise than in summation and squelch, and robust gains have been seen in reverberation when the source of interference is near the subject^11^.

Despite the clear functional benefits yielded by bilateral devices, bilateral implantation is still not popular among adult patients. The cost of a second device and the expenses associated with two surgeries decrease the likelihood of this procedure being offered by health care centers, and particularly public health services such as the Brazilian SUS.

A study on the cost-effectiveness of the cochlear implant procedure for health care services revealed that the clinical benefits provided by bilateral implants in adults were small when the expenditure is considered^12^.

The Digisonic SP^®^ Binaural cochlear implant developed by Neurelec S.A. is an affordable option to bilateral implants, as only one device is used to stimulate both cochleas. This device is priced at a premium of 30% in relation to conventional unilateral implants, and offers benefits equivalent to bilateral cochlear implantation^12^.

The study also indicated a significant squelch effect in Digisonic SP^®^ Binaural device users, with values above the levels observed in users of conventional bilateral implants. This aspect was described by the authors as the outcome of the right/left temporal correspondence allowed by the synchronous stimulation provided by the binaural implant^13^.

The Digisonic SP^®^ Binaural device processes the sound stimuli arriving at both ears in a separate, simultaneous fashion, as also seen in bilateral cochlear implants. However, differently from bilateral devices, the binaural implant offers synchronous electric stimulation between the cochleas. In other words, the device provides stimulation in different frequency bands alternating between cochleas for each sequence of pulses, promoting correspondence between them for each sound stimulus.

Complications in cochlear device implantation occur somewhat frequently and have been a reason for concern in health care centers during the implementation of new surgical approaches. According to the European Statement on Cochlear Implant Failures and Explantations, failure can be divided into six categories: 1 - failure by impact; 2 - sealing failure; 3 - electronic failure; 4 - problems with the electrode set; 5 - others (specify); 6 - no specific reason^14^.

A study conducted on 550 consecutive cochlear device implantation procedures found 92 (6%) complications. Major complications accounted for 8.9% of the procedures, while minor issues were seen in 7.8% of the cases^15^.

Major complications included problems inserting electrodes, misplaced electrodes, damaged electrodes, compressed electrodes, insertion failure, dislocation, flap dehiscence or infection, cholesteatoma, otomastoiditis, facial palsy with sequela, CSF leak, meningitis, and incapacitating otological symptoms. The following minor complications were described: transient peripheral facial palsy, posterior meatal wall injuries, annulus and tympanic membrane injuries, perilymphatic fistula, bleeding, corda tympani nerve injuries, and hematoma^15^.

## CONCLUSION

Bilateral cochlear implants bring several benefits to patients with severe and profound hearing loss. The Digisonic SP^®^ Binaural device is a cost-effective alternative to bilateral implants. The surgical approach described was performed without complications, and the procedure was proven to be easy and safe.
